# Oncolytic virotherapy for metastatic breast cancer – a case report

**DOI:** 10.3389/fonc.2023.1186888

**Published:** 2023-06-07

**Authors:** Benjamin Gesundheit, Alexander Muckenhuber, Yehudit Posen, Ronald Ellis, Philip David Zisman, Harald Schmoll, Christine Weisslein, Jayadeepa Srinivas Raju

**Affiliations:** ^1^ RapoYerape Ltd., Jerusalem, Israel; ^2^ Institute of Pathology, Technical University of Munich, Munich, Germany

**Keywords:** breast cancer, metastatic, oncolytic virotherapy, immunotherapy, abscopal effect

## Abstract

**Background:**

Breast cancer is one of the most common malignancies worldwide and remains incurable after metastasis, with a 3-year overall survival rate of <40%.

**Case presentation:**

A 40-year-old, Caucasian patient with a grade-3 estrogen receptor-, progesterone receptor-, Her2-positive breast tumor and two lung nodules was treated with intramuscular targeted immunotherapy with trastuzumab and oral tamoxifen hormone therapy, together with customized intra-tumoral oncolytic virotherapy (IT-OV) over a 17-month period. PET/CT imaging at 3 and 6 months showed increased primary tumor size and metabolic glucose uptake in the primary tumor, axillary lymph nodes and lung nodules, which were paralleled by a hyperimmune reaction in the bones, liver, and spleen. Thereafter, there was a steady decline in both tumor size and metabolic activity until no radiographic evidence of disease was observed. The treatment regimen was well tolerated and good quality of life was maintained throughout.

**Conclusion:**

Integration of IT-OV immunotherapy in standard treatment protocols presents an attractive modality for late-stage primary tumors with an abscopal effect on metastases.

## 1 Introduction

Breast cancer (BC) is one of the most common cancers worldwide and is the leading cause of cancer-related death among women (1, 2). The rapidly changing treatment regimens have brought to improved outcomes and a steady decline in mortality over time, with local and systemic therapies achieving cure in up to 80% of patients with early-stage tumors. However, an estimated 20-30% of breast cancers eventually metastasize (MBC) (3), and are then generally treated by conventional surgical techniques, accompanied by adjuvant chemo-, radio-, endocrine- and/or receptor-targeted therapies (4, 5). Nevertheless, many of these modalities are limited by off-target toxicities or treatment resistance, and most patients either fail to show adequate clinical responses or quickly relapse (6). The 3-year overall MBC survival rate is 38%, with median survival times of 8 and 36 months on metastatic involvement of the brain or bone, respectively (3). Therefore, new treatment modalities for MBC are critically needed.

Various natural and engineered oncolytic viruses (OV) have shown therapeutic effects on diverse tumor types (7), including refractory and incurable cancers (8). Intra-tumoral OVs (IT-OV) have been shown to be therapeutically more efficient than systemic OVs, apparently due to their high localized concentrations that exert a direct oncolytic effect on tumor cells without undergoing systemic dilution. Furthermore, direct access of IT-OV overcomes preexisting immunity of the tumor tissue (9). Cumulative experience has shown the anti-tumoral effect of various OV strains (10, 11), including against highly aggressive triple-negative BC tumors (12). Relatively easy physical access to the primary BC tumor and axillary lymph node (LN) metastases renders such tumors suitable candidates for IT-OV. Furthermore, IT-OV of MBC lesions may impart abscopal effects on inaccessible and undetected metastatic lesions by activating a specific tumor-targeted cytotoxic immune response (13, 14). The presented case involved administration of individualized IT-OV therapy as an adjunct to standard hormone-modulating therapies to a patient with MBC.

## 2 Case description

**Figure 1:**
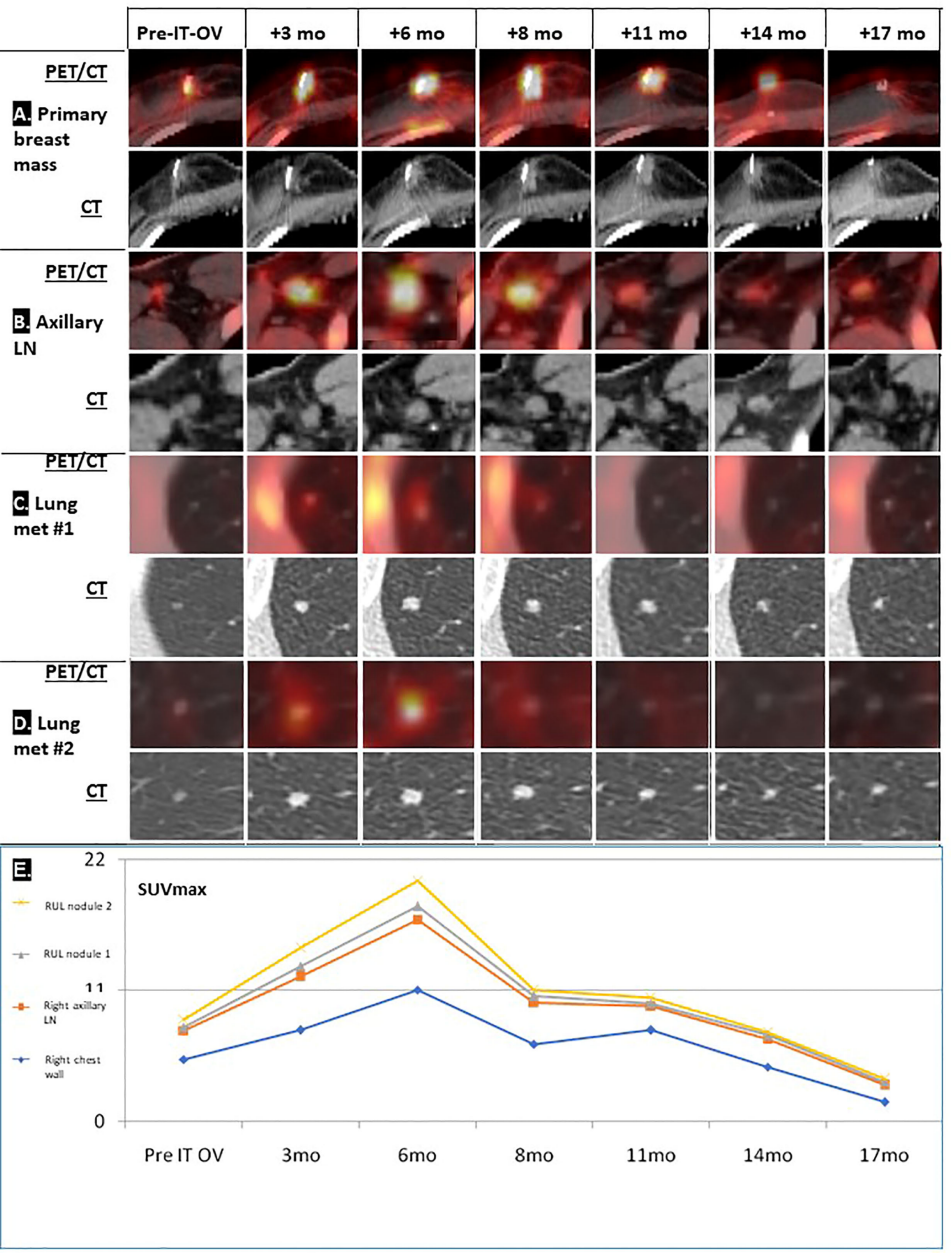
Radiological follow-up of breast lesion and metastases following IT-OV immunotherapy. PET/CT and CT images over the 17 months of treatment of four primary breast masses **(A)** and axillary lymph nodes **(B)** where intratumoral oncolytic virotherapy was administered, and two lung metastases **(C, D)**. Graphic summary of chronological radiological pattern of hyperimmune response in the right chest wall, right axillary lymph node (LN) and right upper lung (RUL) nodules 1 and 2, as measured by PET (SUV_max)_ (see **Figure 2**) **(E)**.

A previously healthy, 40-year-old, Caucasian woman, with no family history of malignancies, reported an indurated right breast mass four months after bilateral silicon breast implantation. Diagnostic MRI showed a 20x17x20mm lesion classified as BI-RADS4. Fine-needle aspiration showed epithelial cell proliferation with suspected atypia. Therefore, the breast lesion was removed. Pathology showed invasive ductal carcinoma with a Nottingham score of 8/9 (glandular differentiation 3/3, nuclear pleomorphism 3/3, mitotic rate 2/3). Due to a suspected residual tumor mass seen in subsequent MRI, a partial right mastectomy was performed to remove the mass with local LNs. Initial pathology showed tumor Grade 3 (G3) without clean margins, no LN involvement (N0), no metastatic spread (M0), positive margins with higher risk (R1), positive estrogen receptors (ER^+^ in 80-90%) and progesterone receptors (PR^+^ in 70-80%), Her-2-neu^+++^ and Ki67 of 80%. Bone scintigraphy showed no bone involvement. Recommended treatments were total mastectomy without the nipple area, followed by four cycles of epirubicin/cyclophosphamide chemotherapy, then 12 cycles of weekly paclitaxel, trastuzumab, endocrine treatment with tamoxifen, zoladex and bisphosphonate. Molecular genetic analysis showed partial resistance to epirubicin and partial-complete resistance to taxanes. Re-assessment by PET/CT showed two sub-centimeter lung nodules in the right upper lobe, thus defining the tumor status as MBC (**Figure 1**).

Based on these findings and limited curative potential with standard treatments, the patient refused aggressive treatments.

## 3 Therapeutic intervention

After signing informed consent for compassionate use of innovative therapies, the patient started intramuscular (IM) trastuzumab 600mg/3-week and oral tamoxifen 20mg/d, and customized IT-OV treatments over a 23-month period (23 sessions) with various combinations of the following OVs at the indicated dosage level per treatment: Newcastle disease virus (NDV, 10^8^-10^9^ TCID_50_), reovirus (REO3, 10^10^ TCID_50_), vaccinia virus (VV, 3x10^8^ TCID_50_), mumps virus (MV, 5x10^7^ TCID_50_), vesicular stomatitis virus 2 (VSV2, 10^9^ TCID_50_) and parvoH1 virus (PH1, 10^7^ TCID_50_). To minimize adverse effects, the first eight sessions were restricted to monotherapy with VSV2, MV, NDV, or REO3. After demonstration of acceptable clinical tolerability, combinations of OVs were administered in subsequent sessions, including NDV-VV, NDV-REO3, NDV-VV-PH1, and NDV-VV-REO3. PET/CT imaging was performed approximately once in 3 months. A summary of the treatment schedule and combinations and imaging schedule is presented in the **Supplementary Table**. Travel strictures during the COVID-19 pandemic resulted in longer treatment intervals, such that higher dosages were administered at later visits. From session 3, Coley’s toxin was intravenously administered at increasing doses to stimulate the innate immune system. From session 5, atezolizumab was intra-lesionally administered in increasing doses (12-24 mg) to enhance immunotherapeutic effects. To maintain tolerability, therapies were adjusted to the following clinical symptoms: hand stiffness for 5 days, transient fever ( ± 39 °C) for several hours with onset <1hr post-IT-OV, swelling of the injected breast and LNs, swelling of the contralateral axillary LNs and the inguinal LN for 3 days, likely reflecting a systemic immune response to the OVs.

**Figure 2:**
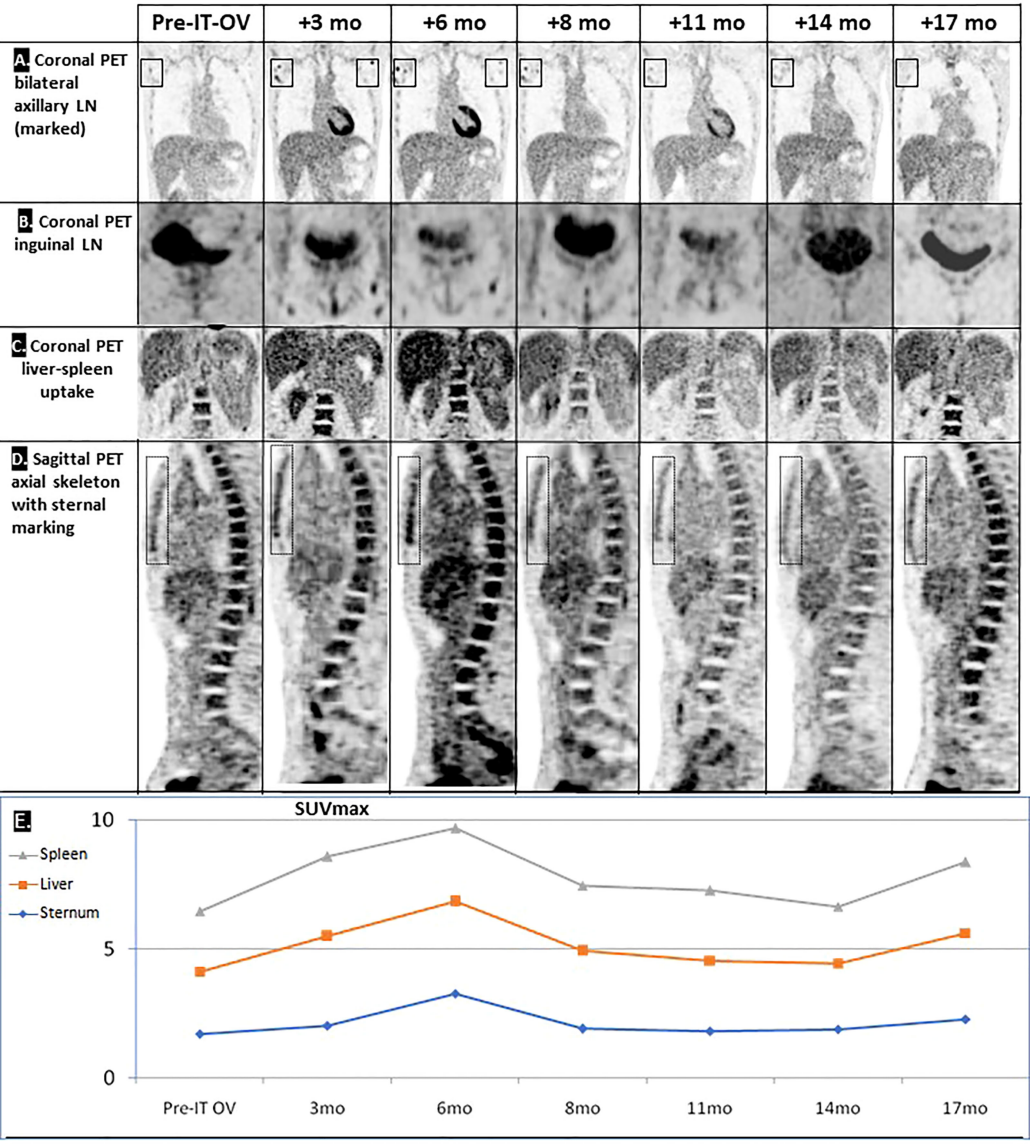
Hyperimmune reaction following IT-OV immunotherapy. Coronal PET showing an apparent OV-stimulated hyperimmune reaction in the local **(A)** and distant **(B)** lymph nodes (LN), in the liver-spleen **(C)**, and in the axial skeleton **(D)**. Graphic summary of chronological radiological pattern of the hyperimmune response, as measured by PET (SUV_max)_ **(E)**.

**Figure 3:**
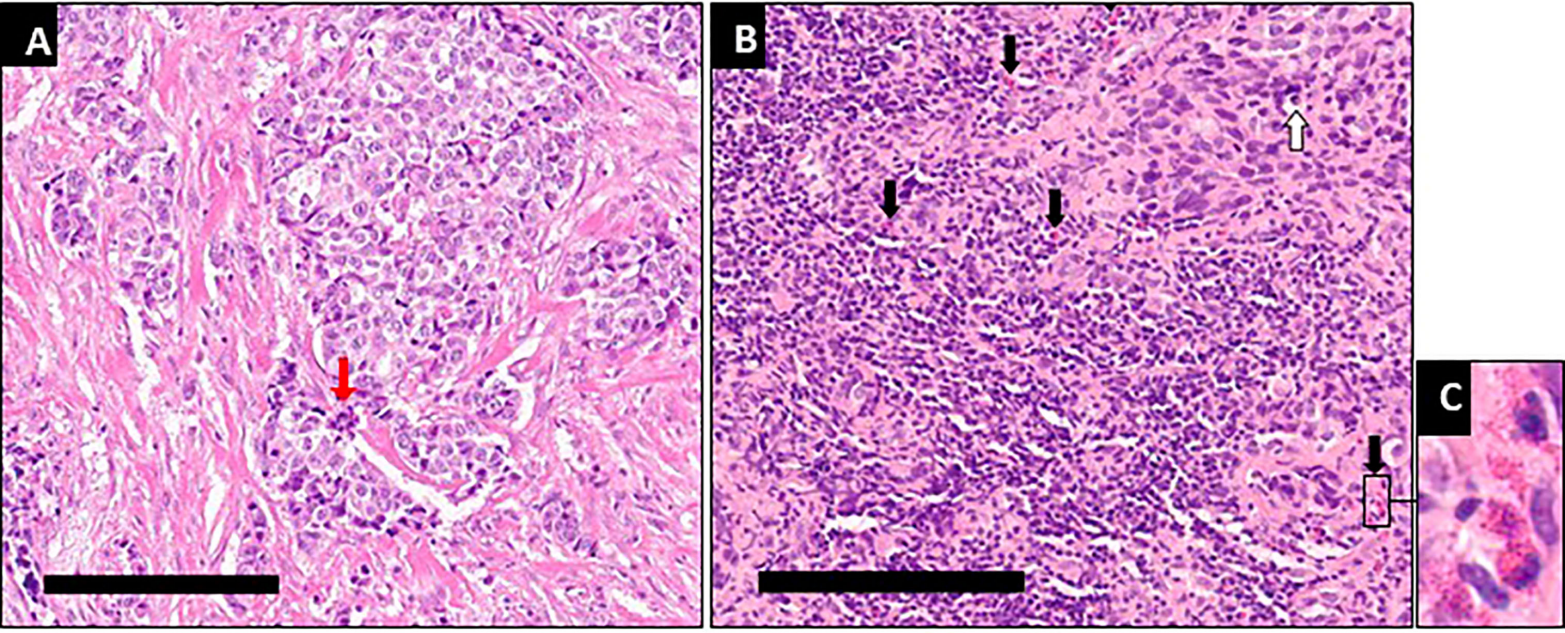
Immunological infiltration and necrotic/fibrotic changes after 9 months of intratumoral oncolytic virotherapy. **(A)** At diagnosis typical features of breast cancer are seen, with proliferation and mitotic cells (red arrows). **(B)** After 14 intratumoral oncolytic virotherapy administrations over 9 months some apoptotic tumor cells (white arrow) are seen along with a pronounced increase in the number of inflammatory cells, primarily in the tumor stroma, mainly lymphocytes and eosinophilic granulocytes (black arrows). Measuring bars equal 200µm. **(C)** Enlarged area from B with typical eosinophilic granulocytes.

Treatment was generally well tolerated, and good quality of life (QoL) was maintained; during the entire course of treatment, no hospital admissions were needed. PET/CT imaging showed typical radiological features of OV-associated immunotherapy (**Figure 1**): After 3 and 6 months, increasing tumor size in CT and metabolic glucose uptake in PET were seen, followed by a steady decline in both parameters resulting radiologically in no evidence of disease (NED) (**Figures 1A, B**). The same response pattern was noted in PET analysis of the metastases, and manifested by increased standardized uptake values (SUV) in both lung nodules, showing mild metabolic uptake in lung nodules which were otherwise too small for PET resolution, likely reflecting a therapeutic abscopal effect (**Figures 1C, D**). Metabolic uptake in adjacent ribs showed the same chronological pattern, presumably reflecting systemic immune activation as seen in the bone marrow (**Figures 1B**, **2**). Comparison of biopsy after 9 months of IT-OV treatments to pre-treatment pathology illustrated infiltration of eosinophilic granulocytes along with some necrotic/fibrotic changes in the tumor mass (**Figure 3**).

Preventive IT-OV with radiological surveillance will be continued.

## 4 Discussion

Our MBC patient had an excellent clinical response to combination therapy with IT-OV, anti-Her2, anti-hormonal therapy, and PD-L1-inhibiting agents and achieved radiologically complete remission. The patient was excited about the curative outcome for her disease, for which the literature gives an overall mean expected survival of 3-5 years. Of no less importance, she retained her fertility. Furthermore, she was pleased with an overall good QoL, no need for hospital admissions nor aggressive treatments like chemotherapy, radiotherapy, mastectomy or thoracotomy.

Given the small size of the lesions, traditional radiological evaluation systems of cancer treatment, e.g., RECIST criteria, were not applicable. Furthermore, the intermittent increase in lesion size and SUV uptake was compatible with pseudo-progression induced by IT-OV injections, attributable to hyper-immune reactions at the cellular level (**Figure 2**). Given these phenomena, radiological monitoring for both anatomic and metabolic parameters are key to accurate assessment of response to OV.

Aside from radiological remission of the treated breast mass and axillary LNs, the combination treatment induced shrinkage and scarring of the two pulmonary metastases in the same chronological and radiological pattern as the primary mass. This was presumably induced by local tumor cell lysis and subsequent release of tumor-associated antigens which stimulate a systemic tumor-directed hyperimmune reaction. Our patient might be the first reported with such an abscopal effect on the pulmonary lesions, similar to the known abscopal responses in melanoma patients (15). The hyperimmune reaction of the skeletal system might have involved immune activation of precursor bone marrow cells (16). The systemic effects of hyperimmune activation have never been reported following trastuzumab or tamoxifen monotherapy and were likely induced by the IT-OV component of the treatment regimen and explain the therapeutic abscopal response of the two lung metastases in the same chronological pattern. This unique phenomenon has promising potential for treatment of distant metastases and justifies investigating this innovative therapeutic approach for MBC in controlled clinical studies.

IT-OV with combinations of various viral strains enhanced tumor cell killing in animal studies (17, 18) and induced systemic anti-tumor immunity, stimulating the innate and adaptive immune responses (19). The choice and regimen of the best OVs and their combinations remain to be explored in clinical research.

Since minimal residual disease cannot be ruled out radiologically, IT-OV with radiological and microscopic surveillance should be continued with interim biopsies to monitor pathological changes and clinical responses. Determination of virus-neutralizing antibody levels and immune markers during treatment should provide valuable insights into optimal scheduling, preferred monitoring techniques, and therapeutic effects of IT-OV. IT-OV combinations with other therapeutic modalities might present a breakthrough for BC/MBC patients by offering significantly improved clinical tolerance, better QoL and most importantly improved therapeutic outcomes.

Limitations of the treatment approach include the lack of generic dosages for all patients; each dose must be customized to the patient’s immune response and later further calibrated in accordance with the imaging and clinical findings. In addition, larger studies covering cases of various degrees of disease burden will be needed to determine the breadth of the effect of oncolytic viruses in this clinical context. Furthermore, the possibility of variations in immune responses across different demographic groups should be evaluated. In addition, there is a possibility that trastuzumab-induced antibody-dependent cell-mediated cytotoxicity would have elicited an adequate response in the patient. However, considering the aggressiveness of mBC and its poor prognosis, a broad combination-therapeutics strategy which relies on multiple mechanisms of action, is expected to enhance prospects for cure.

In the presented case, clinical immune reactions were observed after a combination of IT-OV, anti-hormonal and Her2-targeted treatments, with radiological patterns first showing initial pseudo-progression, followed by gradual tumor shrinkage and eventual cure. This immune activation was also reflected at the precursor bone marrow level in the axial skeleton as a one-time transient phenomenon. These signals are typical for immunotherapy and warrant further investigation into the potential long-range effects of such therapy and its contribution to tumor cure rates.

## Data availability statement

The raw data supporting the conclusions of this article will be made available by the authors, without undue reservation.

## Ethics statement

The studies involving human participants were reviewed and approved by the German Individueller Heilversuch law, which enables physician-driven treatments without requiring a separate ethical committee approval. Written informed consent was obtained from the patient for publication of this case report and any accompanying images. A copy of the written consent is available for review from the corresponding author. The patients/participants provided their written informed consent to participate in this study.

## Author contributions

BG was involved in medical discussions regarding patient care. JSR and BG evaluated the radiological tests. AM prepared the pathology findings. BG, YP, RE, PZ, CW and HS collected and analyzed the data, reviewed the literature and prepared the manuscript.
